# Current research status of HLA in immune‐related diseases

**DOI:** 10.1002/iid3.416

**Published:** 2021-03-03

**Authors:** Bingnan Liu, Yuanyuan Shao, Rong Fu

**Affiliations:** ^1^ Department of Hematology Tianjin Medical University General Hospital Tianjin PR China

**Keywords:** HLA, immune‐related diseases, MHC

## Abstract

Human leukocyte antigen (HLA), also known as human major histocompatibility complex (MHC), is encoded by the HLA gene complex, and is currently known to have the highest gene density and the most polymorphisms among human chromosomal areas. HLA is divided into class I antigens, class II antigens, and class III antigens according to distribution and function. Classical HLA class I antigens include HLA‐A, HLA‐B, and HLA‐C; HLA class II antigens include HLA‐DP, HLA‐DQ, and HLA‐DR; nonclassical HLA class I and II molecules include HLA‐F, E, H, X, DN, DO, and DM; and others, such as complement, are class III antigens. HLA is closely related to the body's immune response, regulation, and surveillance and is of great significance in the study of autoimmune diseases, tumor immunity, organ transplantation, and reproductive immunity. HLA is an important research topic that bridges immunology and clinical diseases. With the development of research methods and technologies, there will be more discoveries and broader prospects.

## 1 INTRODUCTION

Human leukocyte antigen (HLA), also known as human major histocompatibility complex (MHC), is encoded by the HLA gene complex. The HLA gene is located on the short arm of chromosome 6 (6p21.31), covering an area of 7.6 Mb and containing more than 250 genes with different functions,^1^ and is currently known to have the highest gene density and the most polymorphisms among human chromosomal areas. HLA is divided into class I antigens, class II antigens, and class III antigens according to distribution and function. Classical HLA class I antigens include HLA‐A, HLA‐B, and HLA‐C; HLA class II antigens include HLA‐DP, HLA‐DQ, and HLA‐DR; nonclassical HLA class I and II molecules include HLA‐F, E, H, X, DN, DO, and DM; and others, such as complement, are class III antigens.^2^, ^3^ HLA is closely related to the body's immune response, regulation, and surveillance and is of great significance in the study of autoimmune diseases, tumor immunity, organ transplantation, and reproductive immunity.^4^ This article reviews the current research status of HLA in immune‐related diseases.

## 2 AUTOIMMUNE BLOOD DISEASES

Autoimmune diseases account for a large proportion of blood system diseases, among which aplastic anemia (AA) is a typical autoimmune‐mediated pancytopenia disease. In AA patients, the percentage of loss of heterozygosity of the short arm of chromosome 6 (6pLOH) has been shown to reach 13%, significantly higher than that in normal healthy people (0.09%), and 6pLOH has been shown to directly lead to the haploid loss of HLA genes on 6p. The Japanese scholar Katagiri^5^ found that HLA allele deletions in 6pLOH(+) clones were concentrated in four alleles (HLA‐A*02:01, A*02:06, A*31:01, and B*40:02) and that in 6613 Japanese AA patients, the expression of these four alleles was higher than that of other HLA genes. The high expression of these four HLA molecules in blood cells is considered a possible cause of cytotoxic T lymphocytes (CTL) killing. 6pLOH(+) clones evade immune attack due to the absence of HLA haploids and become the source of clonal haematopoiesis in patients with AA after receiving immunosuppressive therapy (IST). In a somatic mutation analysis of granulocytes from AA patients, haematopoietic stem progenitor cells (HSPCs) lacking HLA class I alleles maintained clonal haematopoiesis in AA patients in the absence of driver mutations or telomere shortening; driver mutations did not occur in HSPCs (unless HLA loss occurred after somatic mutations in HSPCs); and 6pLOH may provide HSPCs with somatic mutations a survival advantage, leading to the clonal expansion of mutant HSPCs.^6^

To understand the origin and clinical significance of HLA‐allele‐lacking leukocytes (HLA‐LL) in AA patients due to 6pLOH, a research team used flow cytometry to detect the number and lineage classification of HLA‐LL in 144 patients with AA. The results suggested that the clones that could escape from CTL attacks are haematopoietic progenitor cells (HPCs) instead of haematopoietic stem cells (HSCs). HPCs lack the ability to differentiate into lymphoid cells but can self‐renew to support bone marrow production. Maruyama et al.^7^ proposed that the specific killing of HPCs by CTLs may not play a major role in the occurrence and development of AA but that the main role is the nonspecific inhibition of haematopoietic function by cytokines. Analysis of the response of AA patients with different genotypes to treatment showed that the response rate of HLA‐LL(+)AA patients to IST was significantly higher than that of HLA‐LL(−)AA patients and HLA‐A homozygous patients and that the survival rate without bone marrow failure of untreated HLA‐LL(+)AA patients was also significantly higher than that of HLA‐LL(−)AA patients and HLA‐A homozygous patients.

On this basis, Zaimoku et al. studied the frequency of HLA allele loss in 312 AA patients and found that HLA‐B *40:02 was the allele with the highest frequency of loss. In the granulocytes of AA patients, HLA‐B*40:02 has high‐frequency somatic mutations, which may come from 6pLOH(+) clones or from 6pLOH(−) clones. This result suggests that haematopoietic stem/progenitor cells present antigens to cytotoxic T cells via HLA‐B, which plays a key role in the pathogenesis of AA, and that clonal haematopoiesis in AA comes from immune pressure selection.^8^ In addition, they successfully isolated T cell clones that can specifically kill HLA‐B*40:02‐positive haematopoietic cells from a 6pLOH(+) AA patient.^9^ Furthermore, Espinoza et al. induced pluripotent stem cells (iPSCs) in vitro for gene editing and found that haematopoietic stem cells derived from the iPSCs of AA patients with HLA‐deficiency and wild‐type haematopoietic stem cells showed similar haematopoietic abilities and that iPSC‐HSCs lacking HLA‐B4002 escaped specific T‐cell attack. In this study, iPSCs from AA patients were injected and transplanted into nude mice to create an AA mouse model, and the same results were obtained through in vivo studies.^10^ In a follow‐up study, among AA patients who did not carry HLA‐B*40:02, B*54:01 was one of the three most common HLA alleles in 6pLOH(+) patients (29% [5/17]).^11^ To gain insight into the fundamental mechanism of clonal haematopoiesis caused by HSPCs‐lacking HLA‐B5401, HSPCs derived from iPSCs from a large number of AA patients lacking B5401 in monocytes were studied, leading to results similar to those of the above studies.

Recently, some researchers have used the super‐high resolution, single‐molecule, sequence‐based typing (SS‐SBT) method to detect 6pLOH in a patient with severe AA, and the detection rate for 6pLOH was the highest during granulocyte colony‐stimulating factor (G‐CSF) treatment. This result indicated that most of the neutrophil precursor cells stimulated by G‐CSF had already developed LOH at the site of HLA damage. The SS‐SBT method is a simple next‐generation sequencing (NGS) method that can completely cover HLA alleles.^12^

In addition, recent studies on fetal and neonatal alloimmune thrombocytopenia (FNAIT) have shown that the HLA molecule encoded by HLA‐DRA/DRB3*01:01 is very important in this immune response.^13^ This disease is caused by alloantibodies against human platelet antigen 1a (HPA‐1a). The HLA‐DRB3*01:01 allele has a significant dose‐dependent effect on anti‐HPA‐1a levels, and maternal HLA‐DRB3*01:01 alleles have a significant negative dose‐dependent effect on neonatal platelet counts. HLA‐DRB3*01:01 carrier status is not only an important immune risk factor but also has a significant impact on fetal/neonatal outcomes. The HPA‐1a‐related immunity risk for HPA‐1a‐positive infants born to HPA‐1a‐negative, HLA‐DRB3*01:01‐positive women is 25 times higher than that for infants born to HPA‐1a and HLA‐DRB3*01:01 double negative women. Therefore, the HLA‐DRB3*01:01 classification can be used as a clinical tool for risk stratification of HPA‐1a‐negative pregnant women during consultation.^14^ The relationship between the fetal or neonatal outcome of HPA‐1a‐immunized women and maternal HLA‐DRB3*01:01 carrier status has been verified by prospective and retrospective studies.^15^, ^16^, ^17^ In addition, the effect of the HLA‐DRB3*01:01 allele on the prognosis of the fetus or new‐born may be mediated by different anti‐HPA‐1a antibody levels.^18^ There is a significant correlation between the level of HPA‐1a in pregnant women and the prognosis of the fetus or new‐born.^19^

## 3 CONNECTIVE TISSUE DISEASE

In rheumatoid arthritis (RA), HLA is the most important genetic risk factor, of which the HLA‐DRB1 allele is the most important. HLA‐DRB1 alleles, especially DR4 and DR1, encode the HLA‐DRβ chain. HLA‐DRβ contains 5‐amino‐acid sequence motifs (positions 70–74 share 5‐amino‐acid sequences, namely, QKRAA, QRRAA, and RRRAA), which are called shared epitopes (SEs) and are closely related to RA susceptibility.^20^, ^21^ Anticitrullinated protein antibodies (ACPAs) are important RA disease markers. Patients carrying HLA‐DRB1 alleles with SEs tend to be susceptible to ACPA‐positive RA, and those not carrying HLA alleles with SEs tend to be susceptible to ACPA‐negative RA. In SE‐positive RA patients, the ACPA titer in patients with the DRB1*04 allele is higher than that in patients with the DRB1*01 allele.^22^, ^23^ HLA‐DRB1 encoding SEs not only increase the risk of RA but also increase the risk of early disease onset, more severe bone erosion, and ACPA. Moreover, the correlations between SEs and RA and between SEs and disease severity depend on the gene dose. Carrying 2 alleles encoding SEs is associated with a much higher risk of joint damage than is carrying 1 allele. In addition, the DRβ chains encoded by the alleles DRB1*01:03, DRB1*13:01, DRB1*13:02, and DRB1*04:02 have an opposite protective effect in RA.^24^, ^25^ However, some scholars question the theory that SE‐expressing HLA‐DR molecules of RA patients present specific antigens because comparative analysis found that the eluted peptide antigens of different HLA‐DR molecules expressing SEs have almost no overlapping similar fragments.^26^

HLA is closely related to ethnicity and population in anthropological research, and similar findings have been found in RA research. Among European RA patients, DRB1*04:01, *04:04, *01:01, and *10:01 are the main alleles encoding SEs, and in East Asian patients, the most common SE‐encoding allele is DRB1*04:05. Among the Pima, Tlingit, Yakima, and Chippewa Indians, the SE‐encoding allele DRB1*14:02 is an important genetic risk factor for severe RA.^27^

The genetic inheritance of DR and DQ loci presents high linkage disequilibrium, and RA‐related DRB1 alleles are specifically expressed in haploids with specific DQ loci. For example, SE‐encoding DRB1*04:01 appears in haploids containing DQA1*03‐DQB1*03:01 or DQA1*03‐DQB1*03:02; SE‐encoding DRB1*04:04 participates in the formation of DQA1*03‐DQB1*03:02 haploids.^28^ In addition, differential peptide binding can be found between some HLA‐DQ and SE‐encoding HLA‐DRB1 molecules.^29^ Therefore, the molecular mechanism by which HLA‐SE molecule influence RA may be more complicated than the original SE hypothesis.

Scholars performed DRB1 genotyping of 96 adult onset Still's disease (AOSD) patients and 1026 healthy controls and found that DRB1*15:01 has a disease‐inducing effect and that DRB1*09:01 has a protective effect, suggesting a protective effect of amino acid residues 86 and 98 of the DRβ chain.^30^ In that study, patients with MEFV gene mutations showed susceptibility to DR5‐induced AOSD, while patients without MEFV mutations did not show susceptibility to DR5‐induced AOSD, and DR5 patients often presented AOSD associated with macrophage activation syndrome (MAS). DR5 is a broad‐spectrum antigen serotype and can be further divided into HLA‐DR11, DRB1*11:01, and HLA‐DR12.^31^ Studies on systemic juvenile idiopathic arthritis (sJIA) suggest that there is a strong correlation between HLA‐DRB1*11 and sJIA and that the DRB1 allele is the main risk factor for sJIA.^32^, ^33^ Both sJIA and AOSD are autoinflammatory diseases cause by abnormalities in the innate immune system,^34^ and their clinical features and HLA profile are also similar.

The results reported by Jung et al.^35^ indicated that the proportion of HLA‐DP and HLA‐DR lymphocytes in AOSD patients was significantly higher than that in RA and HC patients, while the proportion of HLA‐DQ cells in AOSD patients was significantly lower than that in RA and HC patients. In patients, the level of HLA‐DP is significantly correlated with the level of lactate dehydrogenase (LDH). The expression of HLA‐DR is positively correlated with interleukin (IL)‐23, the erythrocyte sedimentation rate, and IL‐18. The expression of HLA‐DQ is negatively correlated with white blood cells, hemoglobin, and IL‐17.

Recent studies have shown that HLA class II molecules are related to many diseases, such as autoimmunity, allergies, and various infections,^36^ most of which are related to DQ and DR, with DP having less of an impact. Some scholars have studied the difference between DR and DQ, and the results suggest that HLA‐DQ binds more human epitopes than does DR molecules; HLA‐DQ binds pathogen epitopes that are more similar to human proteins than those bound by DR; HLA‐DQ and HLA‐DR molecules bind to pathogens of different species; and HLA‐DQ molecules that bind to self‐antigenic epitopes play a protective role in autoimmune diseases.^37^ In other studies, there have been similar findings, such as the discovery that unstable HLA‐DQ peptide binding causes autoreactive T cells to escape from the thymus.^38^ However, the main inhibitory effect of DQ in immune homeostasis is still controversial. DQ‐mediated proliferative responses have also been reported in autoimmune diseases and infectious diseases.^39^, ^40^

The 2017 Manhattan plot of Behçet's disease (BD) showed a sharp peak on the short arm of chromosome 6, indicating that there is a close relationship between HLA and BD.^41^ Studies have suggested that HLA‐A26 positivity is a risk factor for the development of BD.^42^ Furthermore, research has been conducted on eye diseases in patients with BD, with the following results: HLA‐A26 carriers had a higher risk of iridocyclitis and retinal chorionitis, especially in men; the frequency of HLA‐A*2601 in patients (37.5%) was significantly higher than that in the control group (14.4%)^43^; and HLA‐A*2601 was considered a possible marker for the prognosis of poor vision.^42^ HLA‐B51 is another important risk factor for BD and ocular diseases.^44^, ^45^ In a study of susceptibility to autoimmune diseases, killer cell immunoglobulin‐like receptor (KIR) was shown to play a very important role; most of its ligands are HLA molecules. KIR participates in the activation and inhibition of natural killer (NK) cells by recognizing HLA class I molecules. HLA‐B51 and other HLA molecules related to BD have a common Bw4 epitope, and the interaction with its receptor KIR3DL1/DS1 plays an important role in the pathogenesis of BD.^46^ Some scholars have studied the relationship between BD and KIR3DL1/DS1 gene functional polymorphism, and the results suggested that HLA‐B51 is a risk factor for BD, HLA‐3/11 plays a protective role, Bw4 and Bw4‐80I are risk factors, but with a lower risk than that for B51,^47^ and the receptor for HLA‐A3 and A11 is KIR3DL2.^48^ HLA‐B27 and B51, which are closely associated with ankylosing spondylitis (AS), can effectively activate KIR3DL1 NK cells, a process that may play an important role in AS. Studies of psoriatic disease and AS have also shown the protective effect of the KIR3DL1*004 allele.^49^, ^50^ In addition, studies have found that KIR2DL2 is highly correlated with the occurrence and development of systemic sclerosis (SSc).^51^

In SSc studies, it was found that the serum HLA‐G (sHLA‐G) levels in SSc patients with human herpesvirus‐6 (HHV‐6) infection were significantly increased and that the production of sHLA‐G in endothelial cells can possibly inhibit angiogenesis in the presence of a viral infection.^52^ Elevated plasma sHLA‐G levels in SSc patients may be a marker of disease severity. Another related clinical study reported that the skin and blood HLA‐G levels in SSc patients were significantly different from those in the control group.^53^ A study of skin tissue from 35 patients with untreated early diffuse SSc found local infiltration of CD4^+^CTLs, causing apoptotic cell accumulation because of restriction by HLA class II molecules, leading to tissue damage and fibrotic reconstruction and subsequently causing abnormal functions. SSc skin biopsies revealed that CD4^+^CTLs accumulated near endothelial tissues, HLA class II molecules were upregulated, and endothelial cells were the target cells for apoptosis and HLA class II upregulation.^54^

IgG4‐related disease (IgG4‐RD) is also a chronic, immune‐mediated fibrotic disease, characterized by tumor‐like inflammatory masses, which tend to accumulate in lacrimal glands, salivary glands, the pancreas, bile ducts, and retroperitoneal tissues. It is also closely associated with HLA.^55^

## 4 OTHER SYSTEMIC IMMUNE DISEASES

In addition to connective tissue diseases, autoimmune diseases are widespread in other systems. In studies of multiple sclerosis (MS), it has been found that in European populations, MS is associated with the HLA‐DR*15 and DQ*06 haplotypes,^56^, ^57^ and in Sardinian populations, MS is associated with the HLA‐A*30, B*18, C*05, and DR*03 extended haplotypes and the HLA‐DR*03 and HLA‐DQ*02 alleles.^58^, ^59^ Melis et al.^60^ applied a statistical entropy method and showed that the HLA entropy in a relapsing‐remitting multiple sclerosis (RRMS) group was significantly higher than that in the control group (*p* = .002). Similar findings were identified for KIR entropy, but the level of significance was low (*p* = .043). There was no significant difference between patients with primary progressive multiple sclerosis (PPMS) and the control group. Combined with an analysis of the HLA and KIR systems, the total entropy of RRMS patients was significantly higher than that of the control group (*p* = .001), while the total entropy of the healthy control group and PPMS patients was not significantly different. Therefore, patients with PPMS may lack susceptible HLA haplotypes or alleles. In addition, the application of this method has also revealed that HLA and KIR are associated with the occurrence and development of type 1 diabetes, Hashimoto's thyroiditis, celiac disease (CD), psoriasis, RA, and systemic lupus erythematosus.

A Japanese study performed KIR gene, HLA Ⅰ and HLA Ⅱ allele analysis on 154 patients with autoimmune hepatitis (AIH) and 201 healthy individuals. The results suggested that the KIR3DL1/HLA‐B Bw4‐80Ile and HLA‐DRB1*04:05‐DQB1*04:01 haplotypes were significantly correlated with AIH. In contrast, KIR3DL1/HLA‐B Bw4‐80Thr and KIR2DL1/HLA‐C2 had significant protective effects. In general, KIR3DL1/HLA‐B Bw4 represents a new KIR/HLA pair associated with a good prognosis of patients with AIH, while KIR3DL1/HLA‐B Bw4‐negative patients may have a poor prognosis. In addition, in ulcerative colitis (UC) research, it was found that KIR2DL1/HLA‐C2 is related to UC.^61^

Hashimoto's thyroiditis (HT) is also a common autoimmune disease. A recent study analyzed thyroid tissue samples from 46 patients. Compared with the control group, the HT group had significantly more HLA class I molecule‐positive samples, and the semi‐quantitative score for HLA class I molecule expression was significantly higher.^62^

Noninfectious uveitis (NIU) is currently considered to be an autoimmune uveitis. It often develops into spontaneous recurrent inflammation and is mediated by CD4^+^ T lymphocytes and proinflammatory cytokines. Crabtree et al.,^63^ in rats, administered a single intravitreal injection of AAV particles containing complementary DNAs encoding HLA‐G1 and HLA‐G5 subtypes, before simulated inducing NIU and then induced experimental autoimmune uveitis (EAU). The clinical and histopathological inflammation scores were significantly reduced in target eye tissues with AAV‐mediated HLA‐G1 and G5 gene expression compared with the contralateral EAU eye without injection. This result suggests that AAV‐HLA‐G1/5‐targeted ocular gene delivery may reduce off‐target risks and can establish long‐term immunosuppression and that AAV‐HLA‐G1/5 gene therapy can reduce the inflammatory response in EAU rats.

Bullous pemphigoid (BP) can be induced by dipeptidyl peptidase‐4 inhibitor (DPP‐4I). DPP‐4I can alter immune regulation by inhibiting DPP‐4, leading to autoimmune diseases. The immunological characteristics of HLA‐DQB1*03:01 may be biomarkers of genetic susceptibility to DPP‐4I‐BP.^64^ HLA‐DQB1*03:01 has a strong correlation with noninflammatory DPP‐4I‐BP in Japanese patients.^65^ In terms of genetic susceptibility, Sun et al.^66^ emphasized the importance of HLA‐DQB1*03:01 in the occurrence and development of BP, as this allele was detected in about half of BP patients, while HLA‐DQB1*03:03 and HLA‐ DQB1*06:01 were alleles with significant protective effects.

CD is a systemic immune disease. The intake of gluten in the diet of susceptible people may trigger the MHC class II molecular heterodimers DQ2 and DQ8, encoded by specific HLA‐DQ allele variants (DQA1*0501‐DQB1*02 and DQA1*0301‐DQB1*0302), in this population.^67^ Some studies have emphasized that the HLA‐DQB1 locus, especially the HLA‐DQB1*02 allele, plays a more important role. Regardless of other HLA‐DQ alleles, diploid HLA‐DQB1*02 is highly correlated with children's CD, and haploid HLA‐DQB1*02 significantly increases the occurrence of CD.^68^, ^69^ A recent study involving gene sequencing and HLA‐DQB1 gene frequency in CD‐susceptible populations in Kazakhstan led to findings similar to those for populations in countries with a high incidence of CD.^70^

In sarcoidosis, there are many characteristic HLA genes that encode class I HLA‐A and B molecules and class II HLA‐DPB1, DQB1, DRB1, and DRB3 molecules. A recent study that sequenced susceptibility sites for sarcoidosis patients found that in Finnish patients with sarcoidosis, the HLA‐DRB1∗03:01 and HLA‐DRB1∗04:01‐DPB1∗04:01 haplotypes suggest a good prognosis.^71^ In addition, some studies suggest that both sarcoidosis and Sjogren's syndrome are associated with the HLA‐DR3 gene.^72^

Current studies suggest that there are immune factors involved in the onset and outcome of Parkinson's disease^73^, ^74^ and that the level of HLA‐DR expression in patient monocytes is closely related to better cognitive function, semantic fluency, and motor function. In contrast, the results of studies that used animal models of Parkinson's disease involving acute toxins, protein injections, and pathological abnormalities indicated that increased HLA‐DR can have harmful effects.^75^, ^76^ Some scholars speculate that it may be because in animal models, effects such as HLA‐DR‐promoted inflammatory factor release have a greater impact than the beneficial effects of antigen presentation and pathological protein clearance; however, in chronic clinical patients, the beneficial effect of increased HLA‐DR in mediating pathological clearance is greater than the harmful effect.^73^

## 5 TUMOR IMMUNITY

Abnormalities in MHC participate in the immune escape mechanism of tumor cells, a mechanism that has been observed in various tumors. Researchers have detected the expression of MHC class I and class II proteins on the surface of tumor cells in 181 untreated melanoma patients, analyzed their transcription levels and genomes, and investigated the relationship between MHC and the clinical response to anti‐CTLA‐4 therapy, anti‐PD‐1 therapy or combined treatment. The results showed that the response to anti‐CTLA‐4 treatment requires melanoma cells to strongly express MHC class I molecules, while the response to anti‐PD‐1 treatment is related to IFN‐γ‐mediated immune activation, including the expression of MHC class II molecules and innate immunity produced when MHC class I molecules are immune compromised.^62^ In a study of autologous dendritic cell therapy for malignant mesothelioma, after patients with mesothelioma received autologous dendritic cell therapy, the expression of HLA‐DR on peripheral CD4^+^ T cells increased.^77^ Studies using the A20 B‐cell lymphoma cell line and the B16 melanoma cell line showed that antigen secretion by tumor cells and the indirect presentation of antigen‐presenting cells play key roles in the rejection of tumor cells by CD4^+^ T cells. MHC class Ⅱ molecules on the surface of tumor cells participate in this process but do not play a critical role.^78^ In addition, studies have shown that the expression of MHC class II transactivator (CIITA) in EB virus‐positive diffuse large B‐cell lymphoma (DLBCL) is significantly reduced compared with that in EB virus‐negative DLBCL and that gene aberrations involving CIITA are more common in EBV + DLBCL.^79^

## 6 INFECTIOUS DISEASES

A study of HBV infection among 1440 Argentines showed that the infection rate in central Argentina was low, while the infection rate in northwest Argentina was moderate; however, there was no obvious genetic difference between healthy and infected people. In the infected persons in these two regions, three SNPs were detected: rs3077 (HLA‐DPA1), rs9277542 (HLA‐DPB1), and rs7453920 (HLA‐DQB2); these SNPs were significantly related to anti‐chronic HBV infection factors and virus clearance factors, and SNP rs2856718 (HLA‐DQB1) was significantly related to susceptibility to chronic HBV infection.^80^

In addition, the results of studies on the correlation between delayed HBeAg seroconversion and immune‐related gene loci in patients with chronic hepatitis B showed that the expression of rs2621377 (HLA‐DOB) and rs3130215 (HLA‐DPB2) was significantly related to delayed spontaneous e antigen seroconversion, of which rs2621377 (HLA‐DOB) was an independent risk factor for delayed spontaneous e antigen seroconversion.^81^ Studies have shown that HLA‐DQB1 and HLA‐DQA1 are associated with early HBeAg seroconversion in children with chronic hepatitis B^82^ and that the genetic variations in the HLA‐DPA1 (rs3077), HLA‐DPB2 (rs9366816), and HLA‐DPB1 (rs2281388 and rs9277535) loci are significantly correlated with HBV infection, especially with HBV breakthrough in children.^83^ In a study of hepatitis C, some scholars have proposed that the genetic variation in HLA‐DO may be crucial to the outcome of hepatitis C virus treatment in the Chinese Han population.^84^

Researchers have studied the relationship among HLA‐B, HLA‐C and KIR genotypes, HIV‐1 infection, and immune reconstitution inflammatory syndrome (IRIS) in tuberculosis and HIV‐coinfected patients. The results showed that KIR2DS2 is associated with an increased risk of tuberculosis; HLA‐B*08 is a protective factor against the onset of tuberculosis; not carrying KIR2DL3 and carrying HLA‐C*07 are related to the antituberculosis ability of HIV‐infected patients; and the increased risk of IRIS is associated with carrying the KIR2DS2 gene, the HLA‐B*41 allele and the KIR2DS1 + HLAC2 gene pair and with not carrying the KIR2DL3 + HLA‐C1/C2 gene pair and KIR2DL1 + HLA‐C1/C2 gene pair.^85^

SNPs in MHCII‐DRB regulatory genes related to the clinical results of malaria in patients infected with Plasmodium have been investigated. The results showed that the GTAT haplotype has high DRB transcriptional activity, leading to increased DRB expression and subsequent interference in antigen expression and T‐cell activation and thereby causing lower levels of parasitaemia than other haplotypes (GTAC and GGGT).^86^ In addition, it is suggested that HLA‐G plays an important role in various parasitic infectious diseases.^87^

In addition, a study of anti‐interferon‐γ antibodies in Southeast Asian populations showed that these antibodies are closely related to HLA‐DR*15:02/16:02 and HLA‐DQ*05:01/05:02 and thus related to infection by invasive non‐tuberculosis mycobacterial infections and intracellular opportunistic pathogens.^88^

## 7 ORGAN TRANSPLANTATION AND REPRODUCTIVE IMMUNITY

Recently, in the field of organ transplantation and reproductive immunity, there have been many studies on nonclassical HLA molecules. For HLA‐E, most studies have focussed on the two most common genotypes (HLA‐E∗01:01 and HLA‐E∗01:03). In addition, Kordelas et al. analyzed soluble HLA‐E (sHLA‐E) and found that reduced sHLA‐E levels in patients after allogeneic haematopoietic stem cell transplantation are associated with severe acute and long‐term chronic graft‐versus‐host disease and lower overall survival rates; however, no association was found with the three most common HLA‐E genotypes (HLA‐E∗01:03/∗01:03, HLA‐E∗01:01/∗01:01, and HLA‐E∗01:01/∗01:03).^89^

HLA‐G is another nonclassical HLA molecule that has been studied frequently. HLA‐G and HLA‐E are expressed on trophoblast cells during pregnancy, and they promote tolerance towards the “semi‐allogeneic” fetus by binding to inhibitory receptors on NK cells.^90^ HLA‐G and HLA‐E interact to establish an immunosuppressive microenvironment, which helps to avoid tumor surveillance.^91^

Studies have shown that curcumin stimulates angiogenesis through vascular endothelial growth factor (VEGF) and increases the expression of HLA‐G in placental trophoblasts during human early pregnancy, thereby promoting cell growth and migration and stimulating angiogenesis.^92^

Tables 1 and 2, respectively, summarize the new research progress on the roles of HLA class I and class II molecules in the occurrence and development of immune‐related diseases. Summarizing the above research progress, HLA is an important research topic that bridges immunology and clinical diseases. With the development of research methods and technologies, there will be more discoveries and broader prospects.

**Table 1:** Genotypes of HLA class I antigens affecting the occurrence and development of immune‐related diseases

Class I	HLA genotypes	Disease
Classical	HLA‐A*0201	Aplastic anemia (AA)
	HLA‐A*0206	
	HLA‐A*3101	
	HLA‐A*4002	
	HLA‐B*5401	
	HLA‐A*2601	Behçet's Disease (BD)
	HLA‐B*51	
	HLA‐A3(protective)	
	HLA‐A11(protective)	
	HLA‐B*27	Ankylosing spondylitis (AS)
	HLA‐B*51	
	HLA‐A*30	Multiple sclerosis (MS)
	HLA‐B*18	
	HLA‐C*05	
	HLA‐B*08(protective)	Tuberculosis
	HLA‐C*07	Immune reconstitution inflammatory syndrome (IRIS) in tuberculosis and HIV‐coinfected patients
	HLA‐B*41	
	KIR2DS1/HLAC2	
	KIR2DL3/HLA‐C1/C2(protective)	
	KIR2DL1/HLA‐C1/C2(protective)	
	KIR3DL1/HLA‐B Bw4‐80Ile	Autoimmune hepatitis (AIH)
	KIR3DL1/HLA‐B Bw4‐80Thr(protective)	
	KIR2DL1/HLA‐C2(protective)	
	KIR3DL1/HLA‐B Bw4(protective)	
	KIR2DL1/HLA‐C2	Ulcerative colitis (UC)
Nonclassical	HLA‐E*0101	Graft‐versus‐host disease (GVHD)
	HLA‐A*0103	
	sHLA‐E	
	HLA‐G	Systemic sclerosis (SSc)
	sHLA‐G	
	HLA‐G1	Noninfectious uveitis (NIU)
	HLA‐G5	
	HLA‐G	Parasitic infectious diseases
	HLA‐G	Gestation

Abbreviation: HLA, human leukocyte antigen.

**Table 2:** Genotypes of HLA class II antigens affecting the occurrence and development of immune‐related diseases

Class II	HLA genotypes	Disease
Classical	HLA‐DRB3*0101	Fetal and neonatal alloimmune thrombocytopenia (FNAIT)
	HLA‐DRB1*0402(protective)	Rheumatoid arthritis (RA)
	HLA‐DRB1*0103(protective)	
	HLA‐DRB1*1301(protective)	
	HLA‐DRB3*1302(protective)	
	HLA‐DRA1*03	
	HLA‐DRB1*0301	
	HLA‐DRB1*0302	
	HLA‐DRB1*1501	Adult onset Still's disease (AOSD)
	HLA‐DRB1*0901 (protective)	
	HLA‐DP	
	HLA‐DR5	systemic juvenile idiopathic arthritis (sJIA)
	HLA‐DRB1*1101	
	HLA‐DR12	
	HLA‐DR15	multiple sclerosis (MS)
	HLA‐DR*06	
	HLA‐DR*03	
	HLA‐DQ*02	
	HLA‐DQB1*0301	Bullous pemphigoid (BP)
	HLA‐DQB1*0303 (protective)	
	HLA‐DQB1*0601 (protective)	
	HLA‐DR	Systemic sclerosis (SSc)
	HLA‐DRB1*04:05	Autoimmune hepatitis (AIH)
	DQB1*04:01	
	HLA‐DQA1*0501	Celiac disease (CD)
	HLA‐DQB1*02	
	HLA‐DQA*0301	
	HLA‐DQB1*0302	
	HLA‐DRB1*0301(protective)	Sarcoidosis
	HLA‐DRB1*0401(protective)	
	HLA‐DPB1*0401(protective)	
	HLA‐DR3	
	HLA‐DR3	Sjogren's syndrome
	HLA‐DPB2	Hepatitis B (HBV) virus infection
	HLA‐DQB1	
	HLA‐DQA1	
	HLA‐DPA1	
	HLA‐DPB1	
	HLA‐DRB	Malaria
	HLA‐DQ*0501	Invasive non‐tuberculosis mycobacterial infections
	HLA‐DQ*0502	
	HLA‐DR*1502	
	HLA‐DR*1602	
Nonclassical	HLA‐DOB	Hepatitis B (HBV) virus infection

Abbreviation: HLA, human leukocyte antigen.

## CONFLICT OF INTERESTS

The authors declare that there are no conflict of interests.

## Data Availability

All the data used to support this study are available from the corresponding author upon request.
